# Supplementary material for: Urolithin M5 from the Leaves of Canarium album (Lour.) DC. Inhibits Influenza Virus by Targeting Neuraminidase
Source: Molecules. 2022 Sep 5;27(17):5724. doi: 10.3390/molecules27175724 (PMC9457573; doi:10.3390/molecules27175724)
Supplement: Supplementary file 1 [file molecules-27-05724-s001.zip › molecules-1888599-supplementary.pdf]

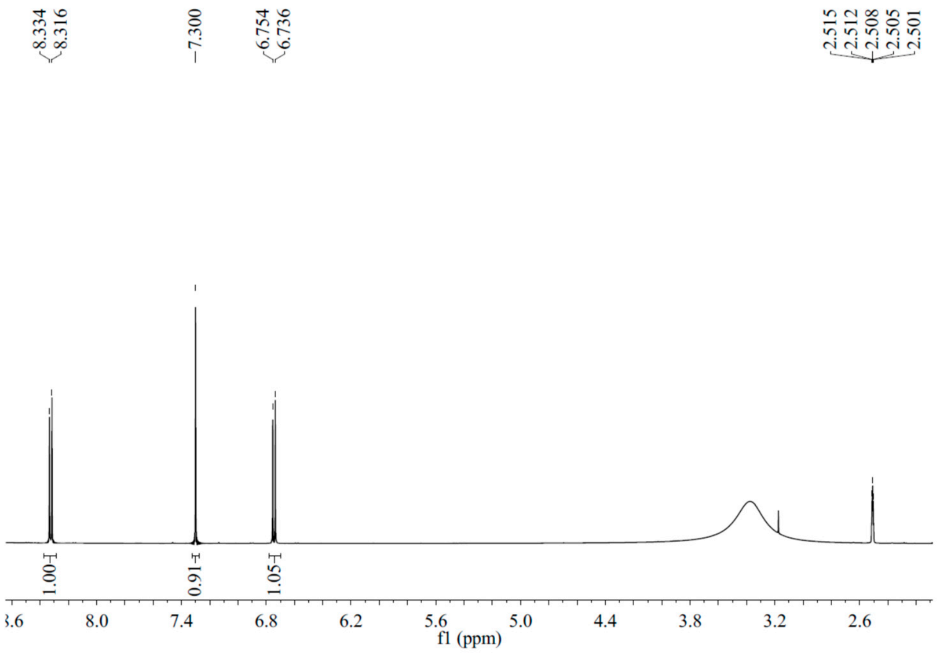

Figure S1. 1H NMR (500 MHz, CD3OD) spectrum of urolithin M5.

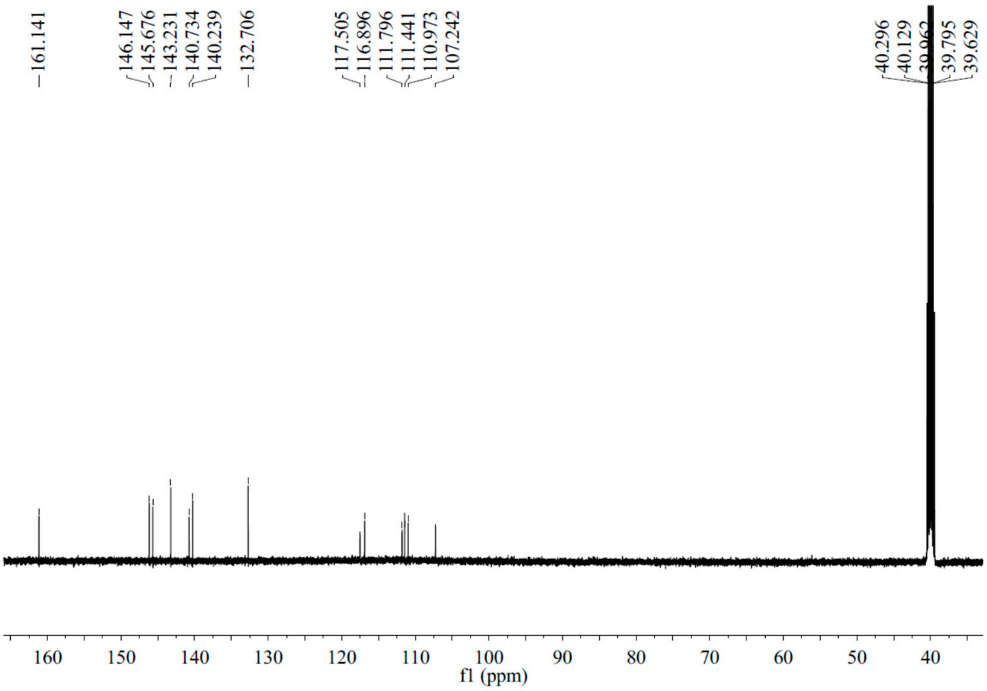

Figure S2. 13C NMR (125 MHz, CD3OD) spectrum of urolithin M5.

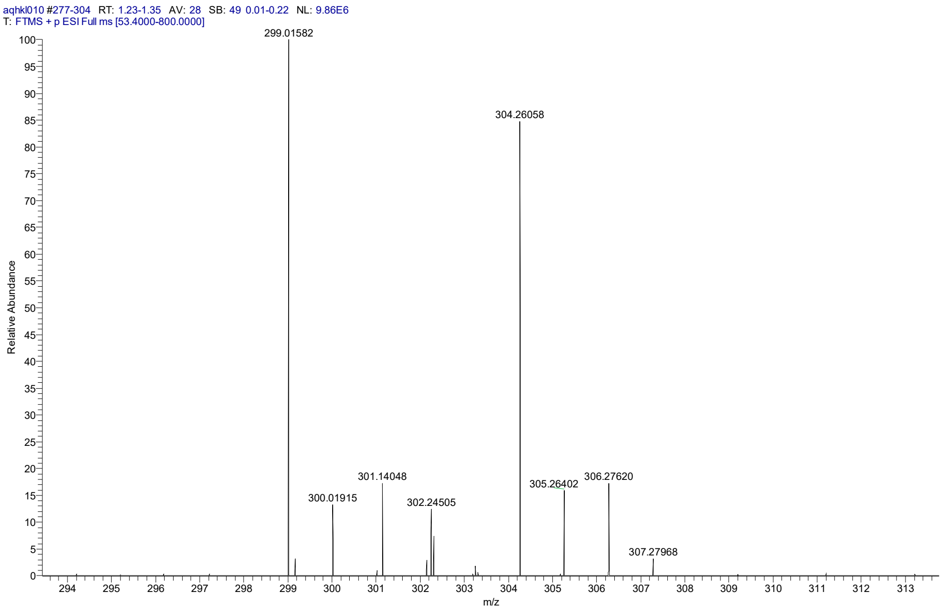

Figure S3. HRESIMS spectrum of urolithin M5.

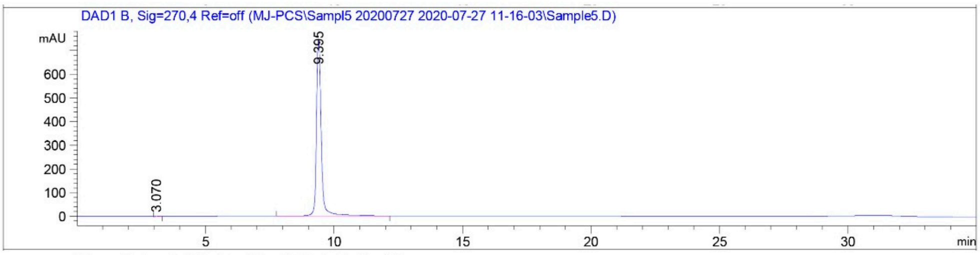

Signal 2: DAD1 B, Sig=270,4 Ref=off

Peak #	RetTime [min]	Type	Width [min]	Area [mAU*s]	Height [mAU]	Area %
1	3.070	BB	0.1359	18.42416	1.99190	0.1897
2	9.395	BB	0.1998	9692.58789	745.11664	99.8103

Figure S4. HPLC spectrum of urolithin M5.

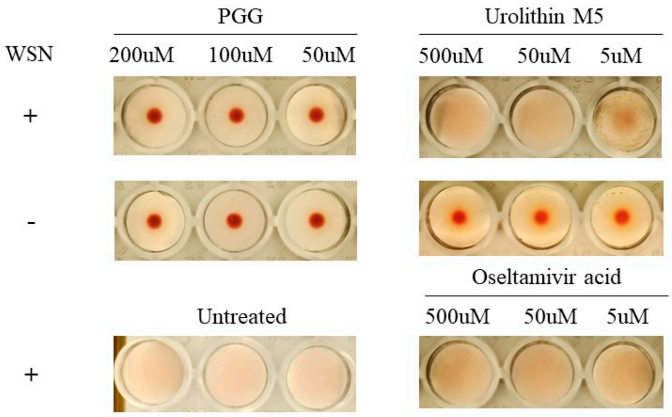

Figure S5. Hemagglutination inhibitory assay of urolithin M5.
